# Dynamics of forage and land cover changes in Teltele district of Borana rangelands, southern Ethiopia: using geospatial and field survey data

**DOI:** 10.1186/s12898-020-00320-8

**Published:** 2020-10-07

**Authors:** Yeneayehu Fenetahun, Wang Yong-dong, Yuan You, Xu Xinwen

**Affiliations:** 1 State Key Laboratory of Desert and Oasis Ecology, Xinjiang Institute of Ecology and Geography, Chinese Academy of Science, Urumqi, 830011 China; 2 National Engineering Technology Research Center for Desert-Oasis Ecological Construction, Beijing, 100049 China; 3 University of Chinese Academy of Science, Beijing, 100049 China

**Keywords:** LULC, Forage biomass, Remote sensing, Teltele, Rangeland, NDVI

## Abstract

**Background:**

The gradual conversion of rangelands into other land use types is one of the main challenges affecting the sustainable management of rangelands in Teltele. This study aimed to examine the changes, drivers, trends in land use and land cover (LULC), to determine the link between the Normalized Difference Vegetation Index (NDVI) and forage biomass and the associated impacts of forage biomass production dynamics on the Teltele rangelands in Southern Ethiopia. A Combination of remote sensing data, field interviews, discussion and observations data were used to examine the dynamics of LULC between 1992 and 2019 and forage biomass production.

**Results:**

The result indicate that there is a marked increase in farm land (35.3%), bare land (13.8%) and shrub land (4.8%), while the reduction found in grass land (54.5%), wet land (69.3%) and forest land (10.5%). The larger change in land observed in both grassland and wetland part was observed during the period from 1995–2000 and 2015–2019, this is due to climate change impact (El-Niño) happened in Teltele rangeland during the year 1999 and 2016 respectively. The quantity of forage in different land use/cover types, grass land had the highest average amount of forage biomass of 2092.3 kg/ha, followed by wetland with 1231 kg/ha, forest land with 1191.3 kg/ha, shrub land with 180 kg/ha, agricultural land with 139.5 kg/ha and bare land with 58.1 kg/ha.

**Conclusions:**

The significant linkage observed between NDVI and LULC change types (when a high NDVI value, the LULC changes also shows positive value or an increasing trend). In addition, NDVI value directly related to the greenness status of vegetation occurred on each LULC change types and its value directly linkage forage biomass production pattern with grassland land use types. 64.8% (grass land), 43.3% (agricultural land), 75.1% (forest land), 50.6% (shrub land), 80.5% (bare land) and 75.5% (wet land) more or higher dry biomass production in the wet season compared to the dry season.

## Background

Rangeland management is a principle involved in regulating and assessing the vegetation, soil, forage production and livestock distribution status [1]. Rangelands represent a key source of pasture for livestock production and covered most of the arid and semi-arid environmental region of Ethiopia [2]. Rangeland vegetation coverage and forage production is determined by both physical (climate, topography and soil) biological (like grazing) and anthropogenic factors [3–5]. The rangelands in Ethiopia including Teltele are rainfall dependent and frequently exposed to irregular rainfall pattern and drought [6]. In addition, this variability has been considered one of the primaries that determined the rangeland forage production [7, 8]. In Teltele, pastoralists rangeland serves as the major economic source [9]. Currently, it is highly vulnerable to the impact of climate change like bush encroachment, drought and the expansion of farming practice that causes decline of forage production and quality [10, 11]. In semi-arid and arid rangeland, climate change and variability determine the structure and function of rangeland vegetation relative to other factors like grazing intensity [12, 13]. The rangeland vegetation status and the forage production also varying spatially with soil characteristics and topography [14]. In Ethiopia, rangelands are lost due to changes in land use system like dramatic expansion of farming practices, establishment of private and government ranches, the rapid infestation of bush plant species and a major facilitator with climate change impact [15, 16]. The Teltele pastoralists typically have a traditional management practice applied to use and manage the resources of their rangeland for a long period of time [17].

However, nowadays, the trend become weakened and part of rangeland replaced by farming are being activated [18]. Managing rangelands requires a combination of biological, physical and social techniques [19]. Unless the management practice cannot be done successfully [20]. Most of the scientific management techniques that have undertaken in Teltele rangeland, have not integrated with the indigenous knowledge of the local community, which has resulted in the practice not succeeding as expected [15, 21, 22]. Today, the degradation of rangeland resource is a serious challenge, bearing negative impact on the pastoral ecosystems, livestock production and people’s livelihoods [23–25]. The main indicator of rangeland degradation includes the decline of total vegetation cover and grass species, increase rates of bush encroachment, depletion of soil quality and reduction of forage quality and quantity [26]. Therefore, a comprehensive analysis of vegetation cover changes and the forage production dynamics that also considers the main driving forces behind these changes is needed to help formulate a sustainable development policy for the rangelands. It is therefore important to evaluate the vegetation cover change and the forage biomass production dynamics so as to recommend appropriate rangeland management techniques. The interpretation of Remotely sensed satellite images in combination with field survey data can be used to evaluate the change in Teltele rangeland. The use of remote sensing techniques for such rangeland change observation, is highly advantageous for to covering a large area with high temporal resolution at low cost, labor intensity and with high accurate and reliable data [27, 28]. Among the different sources of satellite data used for the analysis the rangeland vegetation dynamics, in our study, we used Landsat series [i.e. the multi-spectral scanner (MSS)].

In addition to remote sensing techniques, the evaluation of the impact of the socio-economic dimension on the conversion of rangeland vegetation remains crucial for rangeland vegetation and production change studies [29, 30]. However, there is no scientific report in Telteel rangeland concerning the vegetation change and forage biomass production dynamics in combination of remote sensing data (rainfall data, temperature data) and socio-economic data. Therefore, in this study, we integrated each of above data to assess the changes. The objective of this study is to examine the drivers, trends, and impacts of vegetation change and forage production dynamics in the Teltele rangeland from 1992 to 2019. The specific objectives are: (1) to assess the temporal trends of the expansion of agriculture and shrub encroachment resulting from vegetation changes between 1992 and 2019, (2) to determine the dynamic of forage biomass and relationship with Normalized Difference Vegetation Index (NDVI), (3) to analyze the social economic factors and their influences on the expansion of agriculture and (4) to understand the drivers behind the expansion of agriculture. The rational of this study is to address the major gap connected with information of rangeland vegetation (grass) change to bush encouraged area and cultivated land in Teltele rangeland and valuable for pastoralists livelihood in the district.

## Results

### Classification of vegetation cover change

**Fig. 1:**
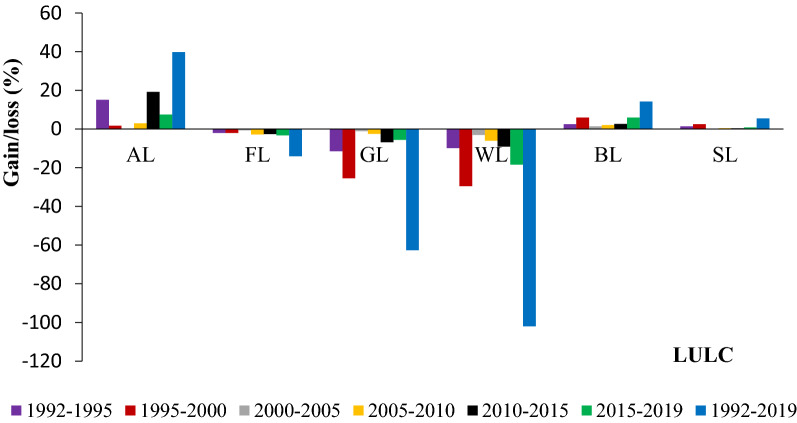
Gain/loss (%) for each LULC between different time periods. *AL* agricultural land, *FL* forestland, *GL* grassland, *WL* wetland, *BL* bare land, *SL* shrub land

**Fig. 2:**
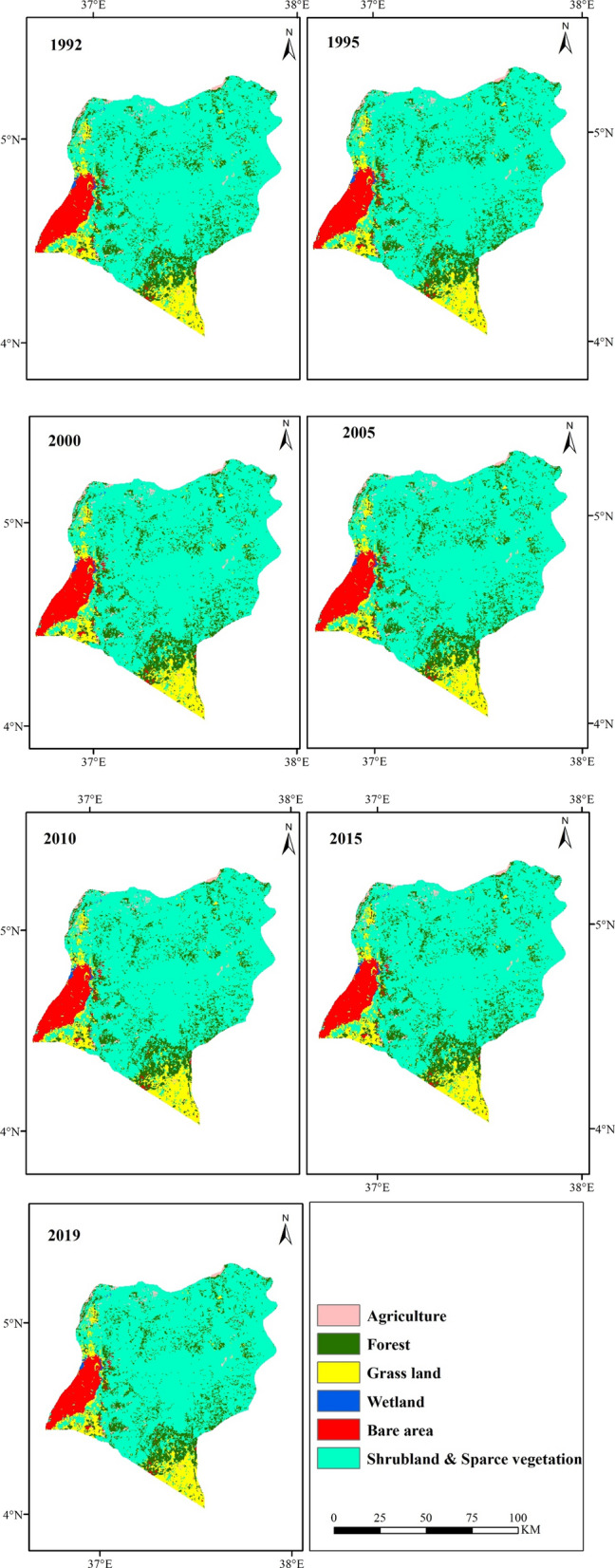
Land cover classes and changes in Teltele rangeland from 1992–2019

The changes of rangeland vegetation cover from 1992 to 2019 are presented in (Table 1; Figs. 1, 2). In order to assess the changes from remote sensing maps, the total number of pixels for each vegetation cover change map (1992, 1995, 2000, 2005, 2010, 2015 and 2019) was recorded. Among the LULC change classes observed in Teltele rangeland, agricultural land, bare land and bush encroachment land increased, while forest, grassland and wetland on rangelands decreased from 1992 to 2019. The agriculture land showed an increasing trend of 15.2% (1992–1995), 1.8% (1995–2000), 0.3% (2000–2005), 3% (2005–2010), 19.3% (2010–2015) and 7.6% (2015–2019) with a net change of 39.8% (1992–2019) (Table 1). The bush land infestation is also one of the challenging issues on the Teltele rangeland and has changed most of the area into un grazing land covered with dense bush. The rate of change was 1.4% (1992–1995), 2.4% (1995–2000), 0.1% (2000–2005), 0.5% (2005–2010), 0.4% (2010–2015) and 0.8% (2015–2019) with the net change of 5.5% (1992–2019). On the side of agricultural land and bush land encroached area, some part of the land has become a non-functional bare area, which means that the covered rangeland has changed to an area which covered neither by grass vegetation nor crop species. The changing rate was high (5.9%) during the period from 1995–2000 and 2015–2019; this is due to the severe impact of the drought (El-Niño) during the year 1999 and 2016 respectively, followed by 2.5% (1992–1995), 1.4% (2000–2005), 2.1% (2005–2010), 2.7% (2010–2015) with a net change of 14.3% (1992–2019).

**Table 1:** Land use/land cover classes and changes from 1992–2019 in Teltele rangeland

LU LC class	Land cover (km^2^)														Changes within periods (%)						
	1992		1995		2000		2005		2010		2015		2019		1992–95	95–00	00–05	05–10	10–15	15–19	1992–19
	A	%	A	%	A	%	A	%	A	%	A	%	A	%	%	%	%	%	%	%	%
AL	98	0.95	115.5	1.1	118	1.15	118	1.1	121.5	1.2	150.5	1.5	162.9	2.5	15.2	1.8	0.3	3.0	19.3	7.6	39.8
FL	1382	13.5	1355.1	13	1328	13.0	1320	12.9	1284	12.5	1251	12.2	1212	11.8	− 2.0	− 2.0	− .6	− 2.8	− 2.6	− 3.2	− 14
GL	1009	9.85	907.6	8.8	724	7.05	715.5	7.0	698.6	6.8	654	6.5	620	6.0	− 11.4	− 25.4	− 1	− 2.4	− 6.8	− 5.5	− 62.7
WL	30.7	0.3	27.6	0.3	21.3	0.2	20.7	0.2	19.5	0.2	18	0.1	15.2	0.15	− 9.8	− 29.5	− 3	− 6.0	− 9.0	− 18.4	− 102
BL	535	5.2	548.8	5.4	583	5.6	591.2	5.8	604	5.9	620.6	6.0	624.3	6.1	2.5	5.9	1.4	2.1	2.7	5.9	14.3
SL	7201	70.2	7301	71	7482	73.0	7490	73	7528	73.4	7561	73.7	7622	74.3	1.4	2..4	0.1	0.5	0.4	0.8	5.5
Total	10,256	100	10,256	100	10,256	100	10,256	100	10,256	100	10,256	100	10,256	100							

*AL* agricultural land, FL forest land, *GL* grass land, *WL* wetland, *BL* bare land, *SL* shrub land. The pixel number change to Km^2^ by using the resolution (300 m) or 0.09 km^2^

Changes between the periods were calculated as: Rate of change (Km^2^/year) = $\frac{{\left( {{\text{A}} - {\text{B}}} \right) \, *{1}00}}{{\text{A}}}$.

where, A = recent land use/ land area in Km^2^; B = previous area of land use/ land cover in Km^2^, and data from 1992 used as a base.

On the contrary, the rangeland area covered with natural forests, grassland and wetlands decreased during the period under study. The loss of grassland, forest and wetlands is mainly attributed to increased agricultural activities, bush infestation and expansion of bare areas, as illustrated on the remote sensing map (Fig. 2; Table 1). Grassland decreased by 11.4% (1992–1995), 25.4% (1995–2000), 1.1% (2000–2005), 2.4% (2005–2010), 6.8% (2010–2015) and 5.5% (2015–2019) with a net change of 62.7% from 1992–2019 (Table 1). Wetland part of Teltele rangeland decreased by 9.8% (1992–1995), 29.5% (1995–2000), 3% (2000–2005), 6% 2005–2010), 9% (2010–2015) and 18.4% (2015–2019) with net change of 102% from 1992–2019. The net change of wetland indicated that more than half (50%) part of the wetland occurred during 1992 change to other land use type either agricultural land, bare land, bush encroached area or others. The natural forest part of the rangeland also decreased by 2% (1992–2000), 0.6% (2000–2005), 2.8% (2005–2010), 2.6% (2010–2015) and 3.2% (2015–2019) with a net change of 14% from 1992–2019.

### Land use and land cover change transition matrix from 1992 to 2019

**Fig. 3:**
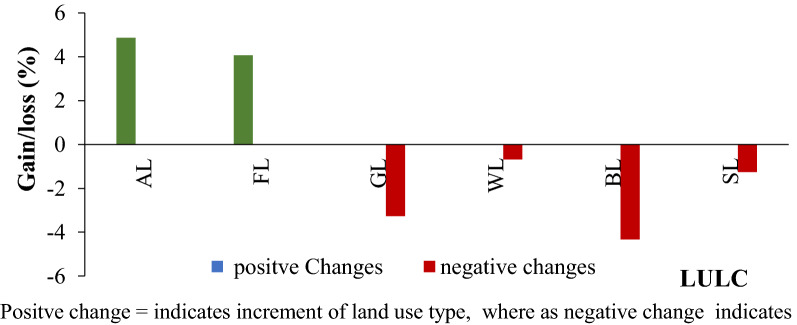
Change percentage of Land use and landcover change transition matrices from 1992–2019. *AL* agricultural land, *FL* forestland, *GL* grassland, *WL* wetland, *BL* bare land, *SL* shrubland

The rate of change trend has shown periodic fluctuations in the Teltele rangeland area. The LULC transitions are the result of either natural factor or human mismanagement of resources during the last almost three decades of the study period. In order to calculate the transition matrix for our case, we overlaid the remote sensing map of 1992 to that of 2019 to generate the matrix which was used to calculate the area of gains, losses and persistence between LULC types [31]. The LULC change directions for the study area from one type to another that have been calculated using Microsoft excel are shown in the transition probability matrix (Fig. 3; Table 2).

**Table 2:** Land use and land cover change transition matrices from 1992–2019

	2019														
	Classes	AL		FL		GL		WL		BL		SL		GT	
		A (km^2^)	A (%)	A (km^2^)	A (%)	A (km^2^)	A (%)	A (km^2^)	A (%)	A (km^2^)	A (%)	A (km^2^)	A (%)	A (km^2^)	A (%)
1992	AL	*96.18*	0.94	0.59	0.006	0.11	0.001	0	0	0.1	0	0.43	0.004	98	1.0
	FL	12.56	0.16	*1246.6*	12.15	0.12	0.001	0	0	58.4	0.57	64.28	0.626	1382	13.50
	GL	7.98	0.08	0.314	0.003	*647.31*	6.31	1.6	0.02	41.93	0.41	309.38	3.016	1009.00	9.8
	WL	8.39	0.08	0	0	5.8	0.056	*16*	0.16	0.11	0.001	0	0	30.7	0.3
	BL	16.31	0.16	0.04	0.003	0	0	0.4	0.003	*520*	5.07	0	0	535	5.2
	SL	9.19	0.1	3.82	0.04	0.66	0.005	0	0	0.18	0.002	*7186.96*	70.07	7201.00	70.2
	G. Total	150.61	1.52	1251.40	12.2	654	6.34	18	0.18	620.72	6.05	7,561.50	73.73	10,256	100

*AL* agricultural land, *FL* forest land, *GL* grassland, *BL* bare land, *SL* shrub land, *WL* wetland, *G. total* grand total, *A* area

The values indicated in bold color across the table indicate that the part of LULC type staying unchanged from one type to another from 1992 to 2019, whereas the rest of the value indicate that LULC changed from one type to another within a time interval from 1992 to 2019. The change detection statistics showed that over 28 years (1992–2019), 35.842% of the grassland, 47.9% of the wetland, 9.8% of the forest land, 1.9% of the agricultural land, 2.8% of the bare area, and 0.2% of the shrub land area were changed to other LULC classes, where we compared the LULC type identified in 1992 with 2019 (Table 2). This indicates that only 64.2% of grassland, 52.1% of wetland, 90.2% of forestland, 97.2% of bare land, 98.1% of agricultural land and 99.8% of shrub land remained within the same LULC types in 2019.

### Forage biomass production dynamics

**Fig. 4:**
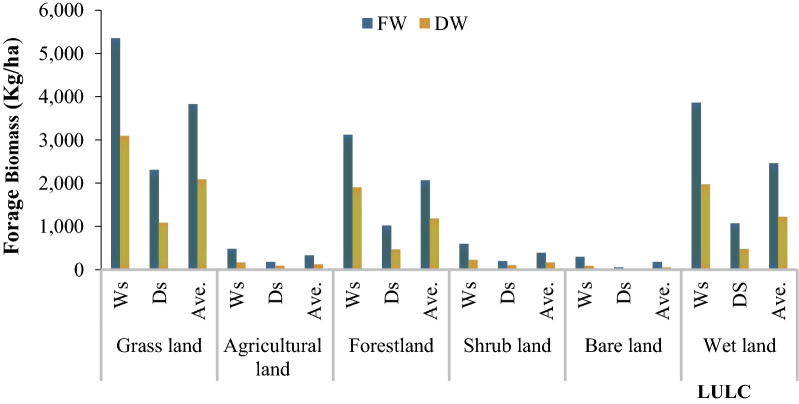
Forage biomass in different land use/cover change both dry and wet seasons*.* *Ws* wet season, *Ds* dry season, *Ave* average, *FW* fresh weight, *DW* dry weight

The present study was tried to assess the biomass production status of forage grass species in Teltele rangeland area using different land cover types. According to the results, the total dry biomass production across all land use types was 3,094 and 1,090.6 kg/ha from grassland, 178 and 101 kg/ha from agricultural land, 1,907 and 475.6 kg/ha from forest land 241 and 119 kg/ha from shrubland, 97.2 and 19 kg/ha from bare land and 1,978.3and 483.7 kg/ha from wetland were recorded during wet and dry season respectively (Fig. 4). When we have seen seasonal impact in all land use types 64.8% (grass land), 43.3% (agricultural land), 75.1% (forest land), 50.6% (shrub land), 80.5% (bare land) and 75.5% (wet land) more or higher dry biomass production during the wet season as compared to the dry season.

### Linkage of forage biomass with NDVI value

**Fig. 5:**
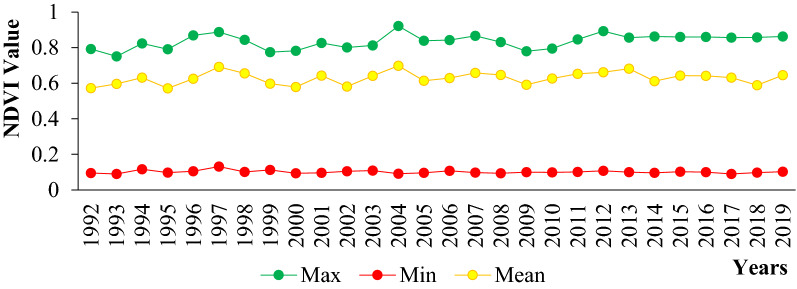
The maximum, minimum and mean annual NDVI value of Teltele rangeland Vegetation index (1992–2019)

The value of NDVI of the general Teltele rangeland showed that, while the annual rainfall was high the NDVI value was high. These values were higher in 2004 (0.8628 and 0.1023) and lower in 2000 (0.7826 and 0.0943) (Fig. 5). The relatively high NDVI value was observed, when the annual rainfall was high and in contrary the annual temperature was low. In addition, if the rainfall pattern was good, the forage biomass production was showed a better result as compared with those years with lower annual rainfall.

**Fig. 6:**
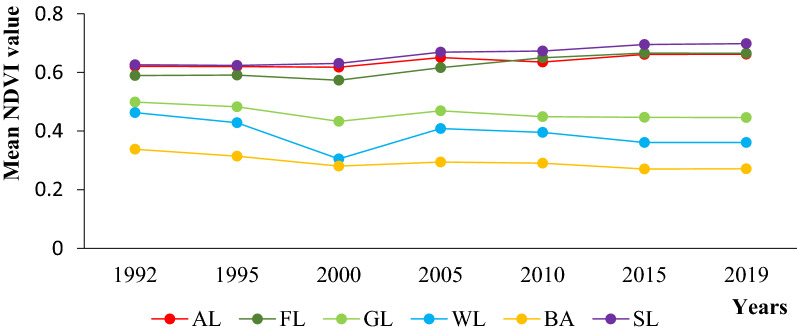
Mean normalized difference vegetation index curves for each vegetation classes*.* *AL* agricultural land, *FL* forest land, *GL* grass land, *WL* wet land, *BA* bare area, *SL* shrub land

The above (Fig. 6), indicates that the NDVI value from 1992 to 2019 has shown both decreasing and increasing trend. The NDVI value of agricultural land, forestland and shrub land showed an upward trend from 1992 to 2019, whereas the NDVI value of bare land, wetland and grassland showed a downward trend. The lowest NDVI value was recorded in 2000 across almost in all LULC types followed with 2019. This is because of the severe drought (El-Niño) occurred in 1999 and 2016 respectively and the scarcity of forage was also observed in this time and this indicates the direct linkage of NDVI value with forage biomass production at certain grazing sites. As we have seen from (Figs. 2, 3; Table 1), the LULC types of agricultural land, shrub land increased due to change in some grassland, forestland and wetland and the NDVI value also showed an upward trend. This told us that the greenery of those areas was better compared with other LULC types from 1992 to 2019. The trend of the NDVI value of agricultural land use type was 0.621(1992), 0.620 (1995), 0.618 (2000), 0.651 (2005), 0.635 (2010), 0.664 (2015) and 0.668 (2019) with a net change in NDVI value increased by 0.047 (6.9%) from 1992 to 2019. The NDVI modified values of Shrub land was 0.626, 0.624, 0.631, 0.669, 0.673, 0.695 and 0.698 for the year 1992, 1995, 2000, 2005, 2010, 2015 and 2019 receptively with a net increase value of 0.072 (11.5%) compared 1992 with 2019. NDVI values of Forest land use also showed an increase value 0.589, 0.591, 0.593, 0.616, 0.650, 0.666 and 0.667 for the year 1992, 1995, 2000, 2005, 2010, 2015 and 2019 respectively with a net increase value 0.078 (13.2%) between 1992 and 2019. This is not related to the forest land, but rather indicates that the forest species found in 2019 showed better greenery performance compared to the forest found in 1992, even though the area coverage declined and changed to other types of land like agricultural land and shrub lands. When we have seen the change in NDVI value for grassland, the wetland showed a downward trend. For grassland the NDVI values were 0.538, 0.517, 0.499, 0.477, 0.463, 0.456 and 0.445 for the year 1992, 1995, 2000, 2005, 2010, 2015 and 2019 respectively with a deceasing NDVI net value of 0.093 (17.3%) as compared 1992 with 2019. The NDVI values of wetland were 0.467, 0.428, 0.305, 0.377, 0.361, 0.353 and 0.354 for the years 1992, 1995, 2000, 2005, 2010, 2015 and 2019 respectively with a decreasing NDVI net value of 0.113 (24.2%). From this we can understand that both the grassland and wetland in Teltele rangeland have been replaced by other land use types and that the grassland greenery and the amount of water have been highly affected by climatic and anthropogenic factors.

### Socio-demographic characteristics of respondents

The gender, occupation and level of education of the respondents were some of the main demographic characteristics that the respondents considered for this study.

**Fig. 7:**
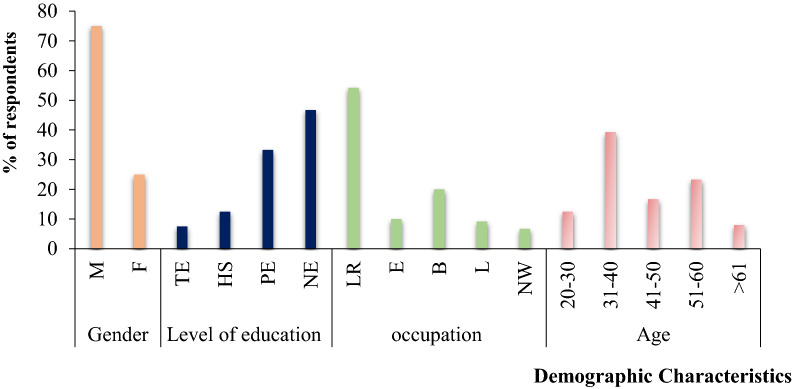
Distributions of respondent’s demographic characteristics. *M* male, *F* female, *TE* tertiary education, *HS* high school, *PE* primary school, *NE* not educated, *LR* livestock rearing, *E* employment, *B* business, *L* labor work, *NW* Not known work

From the above (Fig. 7), we can understand that the gender proportion also took into account and included 75% of males and 25% of females of the total number of participants. This distribution made it possible to understand the perception and coping method dynamics of forage production and of male and female pastoralists. The majority of the respondents (39.2%) were aged between 31–40 years followed by 51–60 years (23.3%) and the age distribution used to evacuate the level of understanding of the general pattern of the study area and the change trend of both forage production and LULC change types during the study period from 1992–2019. Then, the level of education is one of the basic factors on the socio-economic practice within a family and as we have seen from the above figure, the majority of pastoralists (46.7%) were illiterates followed by primary education (33.3%) level. This is because of lack of infrastructure and awareness in the pastoral community based on the data obtained from the respondents and, to some extent, the situation has occurred in the same way with other parts of the country. As a result, most of the livelihood community that depends on livestock occupation has been dispossessed, and this was the major factor causing most of the pastoralists source of income to depend on livestock rearing (54.2%), followed by the management of their own business alongside (20%).

### Driver of change of vegetation land cover and forage biomass

According to the data obtained from group discussion and interviews, the major drivers which influenced the change in land use-land cover and forage biomass production dynamics in Teltele rangeland, the bush infestation ranked as the primary reason (25.8%), followed by drought (20%) and expansion of agricultural practice (15%), increment of the population both human and livestock in the district (12.5%) without additional land provided (Table 3). Government policies have also had their own impact on the livelihoods of pastoralists, in Teltele, which promote the transformation of rangeland into cultivated land and restrict the movement of pastoralists who were traditionally used to coping with the impact of climate change. And also, existence of different insects that eat and damage the forage species (6.7%) and poor pastoralist interaction are also another driving force for change of land use/cover and also forging biomass product in the study area.

**Table 3:** Pastoralist perception related to drivers that cause land use/cover and forge biomass change in Teltele rangeland

No	Driving factors	Number of respondents	Percentage (%)
1	Drought	24	20
2	Increase population number	15	12.5
3	Poor social- interaction	6	5
4	Bush infestation	31	25.8
5	Agricultural expansion	18	15
6	Government policies	13	10.8
7	Insects and disease	8	6.7
8	Gods plan and nature	5	4.2
	Total	120	100

## Discussion

In Teltele the grassland, natural forest and wetland mainly change into agricultural land at an alarming rate. This is due to the factors such as government's current land policies, rapid infestation of bush plant species, climatic and anthropogenic factors. The current land government policies which encouraged the pastoral community to participate in agricultural practice in addition to the scarcity of livestock, since the frequent climatic changes, like drought, challenges the livestock sector and as well as the livelihood of the pastoral community [9, 12]. Therefore, the government encourages the community to reduce their livestock and keep them around their home area by conserving some part of grazing area in the form of ranch and transforming the other part into agricultural [17]. This has caused the degradation of rangeland in the Teltele district. Bush encroachment is another major impacting factor in the study area. The infestation rate was high when the impact of climate change was harsh compared to the other period [32–34]. The most significant change in terrain observed in grassland and wetlands parts of Teltele rangeland occurred during the period from 1995–2000 and 2015–2019, this is due to climate change impact (El-Niño) that happened on the Teltele rangeland in 1999 and 2016 respectively (Figs. 1, 2; Table 1). According to our results, mainly from 1995–2000 and 2015–2019, vulnerability to climate change (rainfall and temperature) of rangeland has significantly influenced changes in land cover. And our result is highly in agreement with the data reported by [35–38].

The frequent decline of water source has a significant impact on the rangeland degradation and also on the livestock population on the rangeland area [34]. The shift of wetland to other land use types like agricultural and bush encroached areas results water scarcity on the grazing area and currently this is one of the major problems in the study area, that caused for the decline of the livestock number [16]. The transition matrices of LULC showed that major change was observed in grassland, wetland and forestland area and mainly transformed to agricultural land, bare land and shrub land LULC types (Table 2) from 2019 year, which is similar to the findings of [39]. As shown in (Fig. 3), the majority of grassland, wetland and forestland have decreased (negative change), whereas the agricultural land, bare land and shrub land have increased (positive change). This indicated that in 2019 (nowadays) the rangeland has degraded due to factors like expansion of farming practices, infestation of bush invasive species, and bare area expansion due to factors like flooding and high wind (climate change) in combination with different anthropogenic factors [40]. The major factors related to the growing challenges of maintaining a livestock-based livelihood system in the face of changing land use and recurring droughts [41]. However, Pastoralists in the study area are conscious of the potential threat of invasive plant species and often, the removal of most shrubs and trees not preferred by livestock on their rangeland had negative impact on the native grass species [35].

Rainfall is the main determinant factor for forage production in all land use type [36, 37]. When we have seen the amount of forage in different land use/cover types, the grasslands had the highest average amount of forage biomass of 2092.3 kg/ha followed by wetland with 1231 kg/ha, forest with 1,191.3 kg/ha, shrub land with 180 kg/ha, agricultural land with 139.5 kg/ha and bare land with 58.1 kg/ha (Fig. 4). This source of variation and dynamics of the forage biomass in the Teltele rangeland is due to a great influence of land use land cover change on the quantity of dry biomass recovered as well as the quantity of fresh weight forage in a given area. Further, the results showed that there was a significant interaction between the season and land cover types with forage biomass production dynamics in the study area and this result is in agreement with [38, 42]. From this we can understand that transition from grazing area (grassland) to other land use type had a significant impact on the reduction of forge biomass production and this was the current major problem on Teltele rangeland resulted to the decline of livestock and scarcity of income in the pastoralist livelihood. The expansion of agricultural land, bush land and bare land area have harmful effects on the forage production dynamics, but the expansion of agricultural land is more problematic in our study site, which is consistent with the data reported by [36, 43]. There is generally a link between the value of aboveground forage greenness (NDVI) and forage biomass and the linkage varied with the season and land use/cover types (Fig. 6). The forage biomass production showed a decline pattern from 1992 to 2019, according to the data obtained from the respondent (Table 5) and filed data (Fig. 4) due to the different driving factors and this is also directly related with the NDVI values. The significant linkage observed between NDVI and LULC changes, was used to estimate the forage biomass production trends across the rangeland compared to each LULC type [43–45]. This implies that there is a direct correlation between rainfall, vegetation greening and biomass production in Teltele rangeland [31]. If the rainfall was high, the vegetation cover of rangeland would be better as compared to the period when the rainfall was low or drought and from this, it can be understood that the greenery was also highly related with the losses of vegetation cover. Our result, directly in line with the data reported by [4]. The overall rangeland vegetation cover analysis and change detection showed remarkable grassland vegetation cover changes across the study site. The greatest change was the decrease in the grassland and wetland proportion of rangeland vegetation and the increase in the cropland and bush infested areas. In general, from the results of the focus group discussion interviews, we can understand that the introduction of privatized resources (enclosures) had caused shortages of communal grazing areas and limited animal mobility.

Thus, the situation affected the socio-economic structure and encouraged pastoralists to diversify their livelihoods with crop production because livestock had become uncertain, which causes the major factor in LULC change of grass land to agricultural land [4]. But there is still a big gap under the term of land use/cover change factors, rather the pastoral community liked that it was due to God's plan and to nature which could be intended to punish us. This was an indicator of community’s low awareness of the climate change with which the whole world is grappling with and our data are consisted with the data reported by [46]. In addition, undermining traditional land use practice also have a direct impact on LULC changes in Teltele [32–34]. Local communities claimed that traditional (customary) laws had become weak and that this has contributed to the observed LULC changes in the area. For example, rotation programs for seasonal grazing areas had been planned and maintained for the specified communities for extended periods [47]. In general, regarding to the Ethiopian's land-use policies and plans, a paper recently presented at the annual World Bank conference noted that because of a lack of a coherent policy use of land, the deterioration of land resources has been documented in the country [48, 49], which is similar to the opinions expressed by the respondents. For sustainable use of pastures in the district of Teltele, an awareness of the pastoral community on land use policies, respectful of the environment and regulating the growth of the human and animal population, was absolutely necessary.

## Conclusions

This study focused on quantifying the status of land use and land cover classes and the forage biomass production of different land use types in Teltele rangeland. A large part of the changes in rangeland vegetation cover differ spatially across the study site. The main characteristics of the LULC changes observed in Teltele rangeland imply a reduction in the total amount of grassland, forestland and wetland, and a significant increase in agricultural, shrub and bare land area. As a result, the forage biomass production also showed great dynamics across each LULC during both wet and dry season. Grassland had the highest average amount of forage biomass of 2092.3 kg/ha, followed by wetland with 1231 kg/ha, forest land with 1191.3 kg/ha, shrub land with 180 kg/ha, agricultural land with 139.5 kg/ha and bare land with 58.1 kg/ha. From this, we can conclude that LULC was the main cause for forage biomass reduction in the Teltele rangeland area. The NDVI value for each LULC type and season showed a direct linkage with forge biomass production and the pattern of change in land use type either negative or loss and positive or gain. The NDVI value showed an increasing trend with land use type of agricultural land, shrub land and also forestland with value of 0.043 (6.8%), 0069 (9.9%) and 0.077(11.6%) respectively, whereas as in the land use type of grassland and wetland NDVI value showed a decreasing trend with value of 0.082 (15.2%) and 0.11(23.6%) respectively. Further, the NDVI value of Teltele rangeland highly related with the rainfall value. The forage biomass production in all LULC types was high, and this related to the greenery of the forage vegetation in the study area. Furthermore, with high NDVI value also high forage biomass production was observed, thus, NDVI and forage biomass have a direct linkage. Therefore, the sustainability of livestock grazing in the district will depend on the health of the grasslands for continuous mobile grazing practices to overcome the low amount of forage per hectare. The driving factors for the LULC changes in the Teltele rangeland area were the bush infestation, drought, expansion of agricultural practice, increment of the population both human and livestock, government policies and insects and disease were mentioned by the respondents. Therefore, land use and management techniques based on the interest of local communities and respectful of the eco-environmental are highly advised and recommended for wise and sustainable use of rangeland resource in the Teltele rangeland. Above all, the balance between the stoking rate with rangeland carrying capacity and balance of the livestock population used to reduce over degradation of the rangeland and the pursuit of awareness will be at the center of priorities.

## Methods

### Study area

**Fig. 8:**
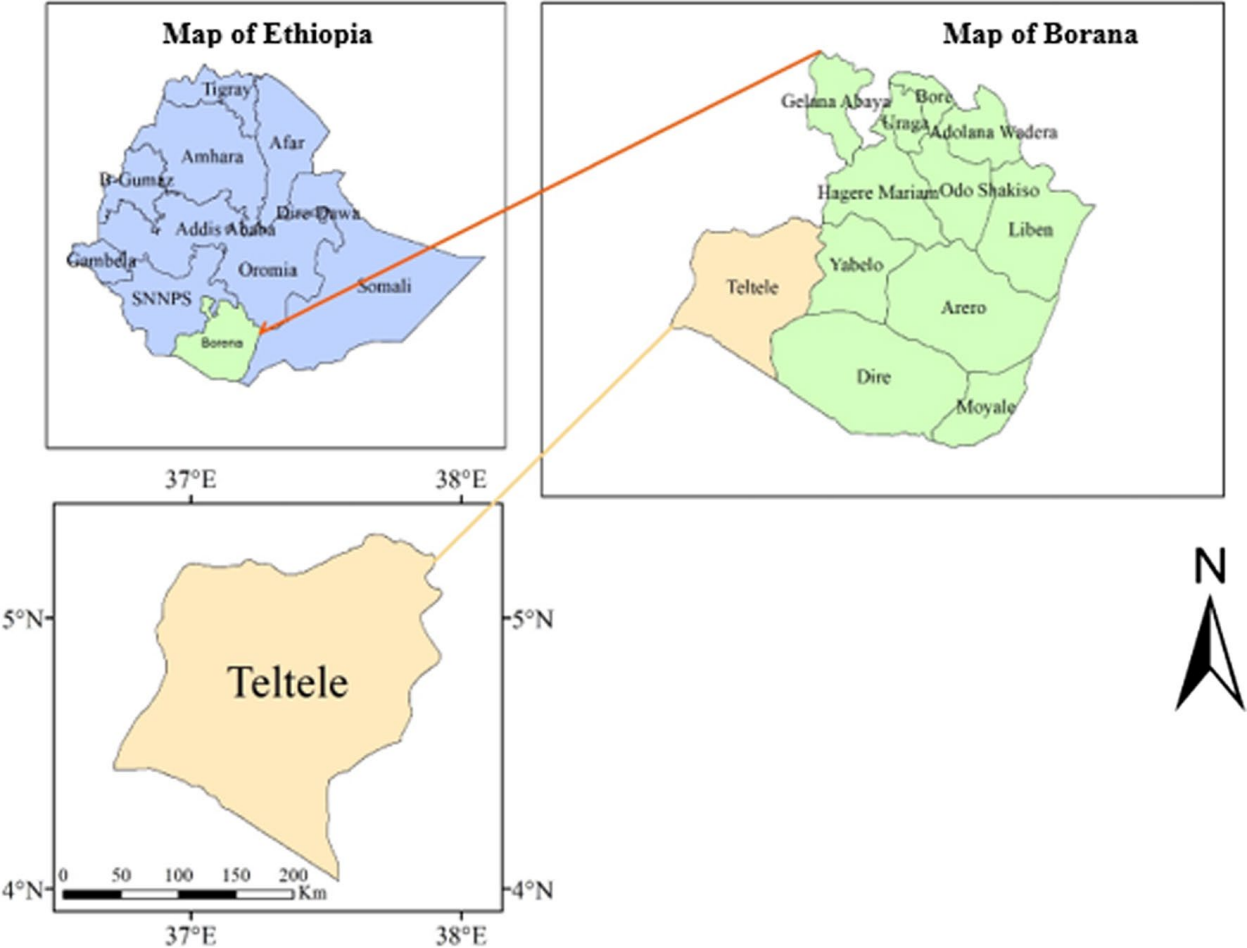
Location map of the study area

**Fig. 9:**
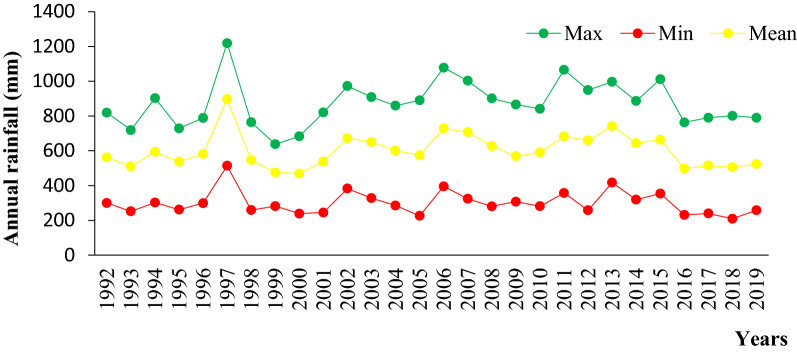
Annual rainfall pattern of Teltele from 1992–2019 (Source: [53]). *Max* maximum, *Min* minimum

The study was conducted at Teltele Woreda in the Borana zone of Southern Ethiopia (Fig. 8). The site was selected because it is one of the most arid parts of Borana zone and, therefore, the pastoral communities of this region are the most vulnerable to the rangeland degradation as a result of both human and climatic factors. It is located 666 km south of Addis Ababa, the capital city of Ethiopia. It lies approximately between 04° 56′ 23′ N latitude and 37° 41′ 51′ E longitude and the altitude are about 496–1500 m, the maximum altitude of 2059 m above sea level. The annual mean temperatures vary from 28 to 33 °C with little seasonal variation. The rainfall in the region is characterized as bi-modal. That is to say that 60% of rainfall occurs from March to May and 27% of rainfall occurs from September to November with high temporal and spatial fluctuations [50] (Fig. 9). The potential evapotranspiration is 700–3000 mm [51]. The soil in the study area includes, 53% red sandy loam soil, 30% black clay, and volcanic light-colored silt clay and 17% silt and the vegetation mainly dominated by encroaching woody species, and those that frequently thinned out, include *Senegalia mellifera*, *Vachellia reficiens* and *Vachellia oerfota* [40, 52]. According to the latest census conducted in 2015, the national census reported a total 70,501 of population for this woreda, of whom 36,246 men and 34,255 women; 4,874 or 6.91% of its population are urban dwellers. Cattle, goats, sheep, camel, mule, donkey and horse are the main livestock species reared.

### Data sources and methods

**Fig. 10:**
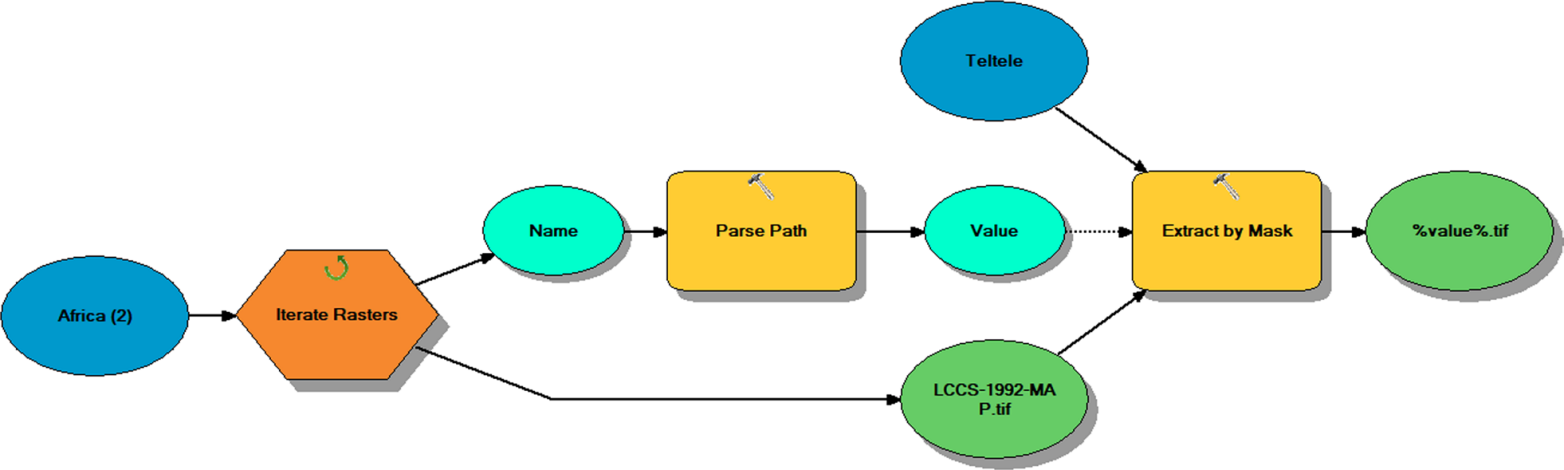
Schematic representation of the model used to extract data to the study area and their bias remove

This study combined multispectral satellite remote sensing data, in-depth fieldwork surveys and rangeland use policy analysis linked with rangeland vegetation change source. The Teltele rangeland shape file along with weather data (rainfall and temperature) from 1992 to 2019 were obtained from [53] to see the long-term trend in the study site. To monitor the spatial and temporal conditions of rangeland vegetation, we used the annual average of third Generation Standard Difference Vegetation Index (NDVI3g) data (1992–2019). The model we used to extract data from the study area and remove the biased from our data in order to adopt land use land cover (LULC) analysis is summarized in (Fig. 10). The data derived from the Global Inventory Modeling and Mapping Studies (GIMMS) with 8 km grid resolution. Before extracting the data to our study area, we resampled them to 300 m resolution of digital elevation model of Ethiopia in order to increase the resolution of the data. For NDVI grid cell values we simply took the maximum, minimum and an average annual mean value in order to reduce disturbance in the trends, such as those attributable to bare soil and sparsely vegetated areas [54, 55]. Vegetation maps of the Teltele district in 1992–2019 were obtained from the remote sensing data with spatial scale 1:100,000. The Landsat TM imageries acquired in 1992, 1995, 2000, 2005, 2010, 2015 and 2019 were used for range land vegetation cover classification and the characteristic of Landsat used for LULC change analysis was described at (Table 4). These years were chosen because of the availability of data, the quality of the images, and in order to compare the changes with in equal time intervals. Further, interviews and focal group discussions were conducted with the local pastoral community and stakeholders to verify the accuracy of the rangeland vegetation classified images analyzed by using ArcMap 10.3.1 software and furthermore, understand the possible major drivers and consequences of LULC changes in the rangeland. A total of 120 individuals (90 males and 30 females), 6 of them were stakeholders from different government sectors (4 males and 2 females) who have been lived 15 to 20 years in the study district, were selected, interviewed and discussed about the rangeland vegetation cover change and forage biomass production trend and as well as the major causes of change based on their observation and experience in the region. The priority driving factors for the changing of rangeland vegetation feature and biomass production were elaborated during the group discussions.

**Table 4:** Characteristic of landsat used for LULC change analysis

Data	Year of acquisition	Bands/color	Resolution (m)	Spectral resolution/bands
Landsat thematic mapper (TM)	1992, 1995, 2000, 2005, 2010, 2015, 2019	Multi-spectral	300	Band 1–5: 0.45–1.75 Band 6: 10.4–12.5 Band 7: 2.08 – 2.35

**Fig. 11:**
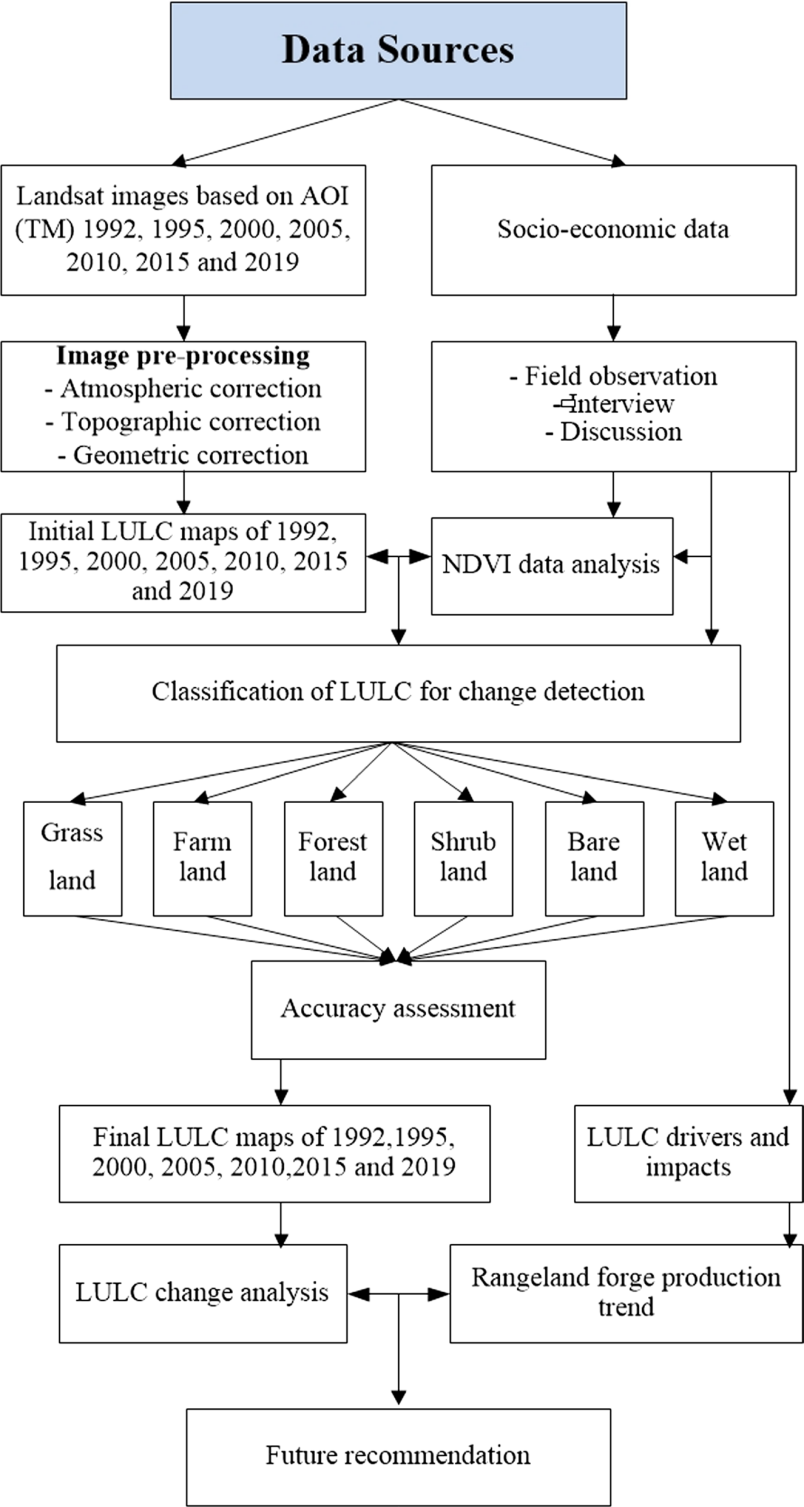
Methodological flow chart of the study

The data pre-processing, clipping the area of interest (AOI) and applying color composites with different reflectance grids, were used to improve visualization and interpretation [56]. The general techniques we used LULC analysis was described in the form of chart below at (Fig. 11).

### Classification of vegetation cover change

In order to clearly understand the change of rangeland vegetation cover, a post classification comparison detection technique was used by classification and detection of each pixel using the remote sensing map and compute the coverage of the area change [57]. The classes were classified based on the Intergovernmental Panel on Climate Change (IPCC) Classes considered for the change detection and Land Cover Classification System (LCCS) Legend used in the Climate Change Initiative Land Cover (CCI-LC) maps for the Images obtained from different years (Table 5).

**Table 5:** Rangeland vegetation change classes and its definition in the study area

No	Class	Definition
1	Grassland	Land cover dominated by grass and herbs
2	Agricultural land	Land area covered with crop fields with rural settlements
3	Forestland	Land covered with higher indigenous plants
4	Shrubland	Bush or shrub-dominated land with small range of grass
5	Bare land	Area neither covered by vegetation nor crops
6	Wetland	Areas seasonally or permanently waterlogged

The most widely used method for change detection is the comparative analysis of the spectral classification over a time series and filtered to reduce the poorly classified pixels [58, 59]. Each classified image was compared for the detection of vegetation cover change and the summaries of the areas and percentages of change were calculated.

### Forage biomass production dynamics

In order to quantify the forage biomass dynamics in different land cover classes, above ground biomass measurement was conducted. A 5 km transect was lied and systematically placed six 25 × 25 m^2^ sampling plot at 500 m interval along a transect at each land class site. (in total 36 plots from the six land classes). In addition, within each plot three (3) 5 × 5m^2^ sub plots (in total 108) were placed. Finally, five (5) 1 × 1 m^2^ quadrants was placed by randomly throwing them backwards in order to minimize any bias resulting from selective placement with in each sub plot for grass species samples collection. Then, all the above ground forage samples were cut by using cutter and collected in paper bag. The fresh weight of forage sample was measured in the field with a scale and taken to Yaballo Pastoral and Dryland Agriculture Research Center soil laboratory and oven dried for 24 h at 105 °C to determine the dry biomass. Then, the dry matter was measured after 24 h drying and converted into kilograms per hectare (kg/ ha). Data collection on grass species sampling was done twice per year (during dry and wet season).

### Determining the linkage between forage biomass and NDVI value

In order to determine the linkage between the forage biomass and the NDVI value, the average NDVI values were derived from plot-specific extractions. The extracted plot-specific NDVI values were matched with the plot-specific forage biomass quantity for each land cover type monitored [60].

### Socio-demographic profile of the respondents

The Social-demographic status (age, sex, education level and income source) of the respondents was analyzed using Microsoft excel and descriptive statistics in the Statistical Package for Social Sciences (SPSS). The spatial and temporal trends in increasing number of agro-pastoralists, the drivers of rangeland vegetation cover and forage production change, the infestation rate of shrub plant species, the expansion agricultural lands, and rangeland indigenous management methods were analyzed using descriptive statistics.

## Data Availability

All the data generated or analyzed during this study are included in this published article and publicly available. The overall data included within this paper was generated by the authors from the satellite data, field collected sample, processing and laboratory analysis.

## Abbreviations

MSS: Multi-spectral scanner
NDVI: Normalized difference vegetation index
LULC: Land use land cover
GIMMS: Global inventory modeling and mapping studies
TM: Thematic mapper
AOI: Area of interest
IPCC: Intergovernmental panel on climate change
LCCS: Land cover classification system
CCI-LC: Climate change initiative land cover
SPSS: Statistical package for social sciences

## References

[1] Allen VG, Batello C, Berretta EJ, Hodgson J, Kothmann M, Li X, McIvor J, Milne J, Morris C, Peeters A, Sanderson M (2011). An international terminology for grazing lands and grazing animals. Grass Forage Sci.

[2] Robin W, Siobhan M, Mark R (2000). Grassland Ecosystem In pilot analysis of global ecosystems.

[3] Han L, Randy A, Dahlgren A, Royce E, Scott M, Devine M, Leslie M, Roche T, Anthony T, Geen O, Andy J, Wong Y, Sarah C, Yufang J (2019). Estimating rangeland forage production using remote sensing data from a small unmanned aerial system (sUAS) and planet scope satellite. Remote Sens..

[4] Michael E, Oliver H, Uwe R, Christian H, Brigitte K, Oliver W (2015). Land conversion dynamics in the Borana Rangelands of Southern Ethiopia: an integrated assessment using remote sensing techniques and field survey data. Environment.

[5] George M, McDougald N, Dudley DM, Connor M, Flavell DK, Vaughn CE, Forero LC, Frost B, Oneto SR (2016). Annual range forage production. Univ Calif Agric Nat Resour Publ.

[6] Anteneh B, Zewdu KT (2016). Mechanisms of bush encroachment and its inter-connection with rangeland degradation in semi-arid African ecosystems: a review. J Arid Land.

[7] McKeon GM, Stone GS, Syktus JS (2009). Climate change impact on Northern Australian rangeland livestock carrying capacity: a review of issues. Rangeland J.

[8] Mei Y, Ellis JE, Epstein HE (2004). Regional analysis of climate, primary production, and livestock density in Inner Mongolia. J Environ Qual..

[9] McCarthy M, Kamara A, Mirk M. The Effect of Environmental Variability on Livestock and Land use Management; The Borana Plateau, Southern Ethiopia. Socio-economic working paper. 35. ILRI, Nairobi, Kenya; 2002.

[10] Fenshamn RJ, Fairfax RJ, Archer SR (2005). Rainfall, land use and woody land cover change in semi-arid Australia Savanna. J Ecol.

[11] Gadzirayi CT, Mutandwa E, Mupangwa JE (2007). Veld condition trend of grazing areas. Why poor livestock production in the tropics?. Rangelands..

[12] Tolera A, Abebe A. Livestock production in pastoral and agro-pastoral production systems of southern Ethiopia. Livest. Res. Rural Dev. 2007; 19: 177. https://www.lrrd.org/lrrd19/12/tole19177.htm. Accessed 29 Dec 2014.

[13] Solomon TB, Snyman HA, Smit GN (2007). Cattle-rangeland management practices and perceptions of pastoralists towards rangeland degradation in the Borana zone of southern Ethiopia. J Environ Manage.

[14] Ayana A, Gufu O, Adunya T (2012). Bush encroachment control demonstrations and management implications on herbaceous species in Savannas of Southern Ethiopia. Trop Sub trop Agroecosyst.

[15] White RP, Wanasselt W (2000). Grasslands in Pieces: Modification and Conversion Take a Toll.

[16] Garedew E. Land Use and Land Cover Dynamics and Rural Livelihood Perspectives in the Semi-Arid Areas of Central Rift Valley of Ethiopia. Ph.D. Thesis. Swedish University of Agricultural Sciences, Umeå, Sweden; 2000.

[17] Bikila N, Bedasa E, Samuel T, Barecha B, Jaldesa D, Nizam H (2014). Control of bush encroachment in Borana zone of southern Ethiopia: effects of different control techniques on rangeland vegetation and tick populations. Pastoralism.

[18] SeleshiY ZU (2004). Recent changes in rainfall and rainy days in Ethiopia. Int J Climatol.

[19] Cheng D, Peili S, Xianzhou Z, Ning Z, Xi C, Wanrui Z (2017). The Rangeland Livestock carrying capacity and stocking rate in the Kailash Sacred Landscape in China. J Resour Ecol.

[20] Tache B. Participatory impact assessment of drought reserve areas in Guji, Borana zone. Report prepared for save the Children’s USA; 2010.

[21] WRI. Drylands, people, and ecosystem goods and services: A Web-based geospatial analysis. In: White RP, Nackoney J (Eds.), World Resources Institute. 2003; 1–58. https://www.wro.org.

[22] Safriel U, Adeel Z, Niemeijer D, Puigdefabregas J, White R, Lal R, Wilson M, Hassan R, Scholes R, Ash N (2005). Dry land systems. Ecosystems and human well-being: current state and trends.

[23] Tsegaye D, Moe SR, Vedeld P, Aynekulu E (2010). Land-use/cover dynamics in Northern Afar rangelands, Ethiopia. Agric Ecosyst Environ.

[24] Turner BL, Steffen W, Jäger J, Carson DJ, Bradshaw C (2002). Toward integrated land-change science: advances in 1.5 decades of sustained international research on land-use and land-cover change. Challenges of a Changing Earth.

[25] Wasonga VO, Nyariki DM, Ngugi RK (2011). Assessing socio-ecological dynamics using local knowledge in the semi-arid lowlands of Baringo districts. Kenya Environ Res J.

[26] Reid RS, Serneels S, Nyabenge M, Hanson J. The changing face of pastoral systems in grass-dominated ecosystems of eastern Africa. In: Suttie JM, Reynolds SG, Batello C, Eds. FAO Grasslands of the world.2005; 19–65.

[27] Brink AB, Bodart C, Brodsky L, Defourney P, Ernst C, Donney F, Lupi A, Tuckova K (2014). Anthropogenic pressure in East Africa Monitoring 20 years of land cover changes by means of medium resolution satellite data. Int J Appl Earth Obs Geoinf.

[28] Brinkmann K, Dickhoefer U, Schlecht E, Buerkert A (2011). Quantification of aboveground rangeland productivity and anthropogenic degradation on the Arabian Peninsula using Landsat imagery and field inventory data. Remote Sens Environ.

[29] Lambin EF, Geist HJ (2006). Land-use and land-cover change: local processes and global impacts IGBP Series.

[30] Pisanelli A, Chiocchini F, Cherubini L, Lauteri M (2012). Combining demographic and land-use dynamics with local communities’ perceptions for analysing socio-ecological systems: A case study in a mountain area of Italy. Forest..

[31] Asnake Y, Amare B (2019). Land use/cover spatiotemporal dynamics, driving forces and implications at the Beshillo catchment of the Blue Nile Basin, North Eastern Highlands of Ethiopia. Environ Syst Res.

[32] Shiferaw H, Schaffner U, Bewket W, Alamirew T, Zeleke G, Teketay D (2019). Modelling the current fractional cover of an invasive alien plant and drivers of its invasion in a dryland ecosystem. Sci Rep.

[33] Tessema K, de Boer F, Prins H (2016). Changes in grass plant populations and temporal soil seed bank dynamics in a semi-arid African savanna: implications for restoration. J Environ Manag.

[34] Tilahun M, Birner R, Ilukor J. Households' demand for mitigation of Prosopis juliflora invasion in the Afar region of Ethiopia: a contingent valuation. Manag. Prosopis Juliflora Better Pastor. Livelihoods Horn Africa Proc. Reg. Conf. May 1–May 12, Addis Ababa, Ethiop; 2014; 0568 10.1080/09640568.2016.1152955.

[35] Haftay H, Yayneshet T, Animut G, Treydte AC (2013). Rangeland vegetation responses to traditional enclosure management in eastern Ethiopia. Rangeland J.

[36] Abdullah M, Rafay M, Sial N, Raseed F, Nawaz M, Nouman W, Ahmad I, Ruba T, Khalil S (2017). Forage productivity, carrying capacity and palatability of browse vegetation in arid rangelands of Cholistan desert (Pakistan). Appl Ecol Environ Res.

[37] Angerer JP (2008). Technologies, tools and methodologies for forage evaluation in grasslands and rangelands.

[38] Habtamu T. The impact of changes in land use patterns and rainfall variability on range condition and pastoral livelihoods in the Borana rangelands of Southern, Ethiopia. A PhD Thesis. University of Pretoria, South Africa; 2013.

[39] Hurgesa H, Sylvester M, Amare B (2019). Spatiotemporal analysis of land-use and land-cover dynamics of Adama District, Ethiopia and its implication to greenhouse gas emissions. Integr Environ Assess Manag.

[40] Coppock DL (1994). The Borana plateau of southern Ethiopia: Synthesis of pastoral research, development and change. Livestock Center Afr.

[41] Fikre Z, Abdurhman M (2019). Land Use and Land Cover Dynamics in Eastern Pastoral Rangelands of Somali Region. Ethiopia. J Environ Earth Sci..

[42] Miehe S, Kluge J, Wehrden H, Retzer V (2010). Long-term degradation of the Sahelian rangeland detected by 27 years of field study in Senegal. J Appl Ecol.

[43] Bai ZG, Dent DL. Global assessment of land degradation and improvement: A pilot study in Kenya. World Soil Information Report; **2**006/1; https://www.isric.org/isric/webdocs/docs/ISRIC_Report_2006_01.pdf.

[44] Bozkurt Y, Uzun N, Dogan C (2011). Grassland evaluation based on GIS model and remote sensing data for beef cattle grazing. Grassland Science in Europe.

[45] Dwyer PC. Spatial estimation of herbaceous biomass using remote sensing in Southern African savannas, MSc. Thesis. Johannesburg: University of Witwatersrand; 2011.

[46] Han J, Zhang Y, Wang C, Bai W, Wang Y, Han G, Li L (2008). Rangeland degradation and restoration management in China Rangeland J.

[47] Alemu B, Garedew E, Eshetu Z, Kassa H (2015). Land use and land cover changes and associated driving forces in north western lowlands of Ethiopia. Int Res J Agric Sci Soil Sci.

[48] Gebeyehu ZD, Woldegiorgis SB, Belete AD, Abza TG, Desta BT. Ethiopia’s move to a national integrated land use policy and land use plan. In: Proceedings of the 2017 World Bank Conference on Land and Poverty. Washington DC; 2017; P. 28 . https://www.landlinks.org/wpcontent/uploads/2017/03/USAID_Land_Tenure_WB17_Ethiopia_17. Move_Land_Use_Plan.pdf.

[49] Gamoun M (2014). Grazing intensity effects on the vegetation in desert rangelands of Southern Tunisia. J Arid Land..

[50] Dalle G, Maass BL, Isselstein J (2015). Rangeland condition and trend in the semi-arid Borana lowlands, southern Oromia, Ethiopia. Afr J Range Forage Sci.

[51] Billi P, Alemu YT, Ciampalini R (2015). Increased frequency of flash floods in Dire Dawa, Ethiopia: Change in rainfall intensity or human impact?. Nat Hazards.

[52] Gemedo D, Maass BL, Isselstein J (2005). Plant communities and their species diversity in the semi-arid rangelands of Borana lowlands, southern Oromia. Ethiop Commun Ecol.

[53] Ethiopian Meteorological Agency (2015). Recorded rainfall and temperature data of Teltele Districts in Borana Rangeland; Ethiopian meteorological agency: Addis Ababa.

[54] Slayback DA, Pinzon JE, Los SO, Tucker CJ (2003). Northern hemisphere photosynthetic trends 1982–1999. Glob Chang Biol.

[55] Wang XH, Piao SL, Ciais P, Li GS, Friedlingstein P, Koven C, Chen A (2011). Spring temperature change and its implication in the change of vegetation growth in North America from 1982 to 2006. Proc Natl Acad Sci USA.

[56] Lillesand TM, Keifer RW, Chipman JW (2008). Remote Sensing and Image Interpretation: Digital Image Interpretation and Analysis.

[57] Anderson JR, Hardy EE, Roach JT, Witmer RE (1976). A land use/cover classification system for use with remotely sensing data; us geological survey professional paper 964: Sioux Falls.

[58] Singh RB, Fox J, Himiyama Y (2001). Land use and land cover change; Science Publishers: Enfield.

[59] Charles A, Yuji B (2009). Analysis of land use/cover changes and animal population dynamics in a wildlife sanctuary in East Africa. Remote Sens.

[60] Anthony E, Oliver W, Joseph K, Mwanjalolo M, Laban M, John M (2014). Spatio-temporal dynamics of forage and land cover changes in Karamoja sub-region, Uganda. Pastoralism..

